# A rare ultrasound case report: intramuscular tear of the sartorius muscle

**DOI:** 10.1186/s13089-019-0132-9

**Published:** 2019-07-26

**Authors:** Kathryn Malherbe

**Affiliations:** Diagnostic Imaging Solutions, Pretoria, 0001 South Africa

**Keywords:** Ultrasound, Sartorius muscle, Intramuscular tear, Intrinsic muscle injury

## Abstract

We report a case of a 65-year-old male patient who presented with localized swelling and lump in the proximal third of the upper thigh. Most common upper thigh injuries involve the quadriceps muscle group associated with football injuries; however, in rare incidents certain forceful intrinsic injuries can cause intramuscular tear of the sartorius muscle. This is a rare case of spontaneous muscle rupture in an elderly patient with no history of recent sporting activity. Ultrasound provides a cost-effective, dynamic evaluation of the anterior upper thigh muscles to differentiate between chronic and acute phases of injury.

## Introduction

Muscle fibres are grouped into fascicles and separated by septa of fibro-adipose tissue, the perimysium. The muscle is enclosed by a fascial sheath, the epimysium. The perimysium is seen on transverse imaging as dot echoes throughout a hypoechoic background, representing the bulk of the muscle fibres. Intramuscular septa produce a reticular pattern, and intermuscular septa appear brightly echogenic [1].

The sartorius muscle (Latin-sator, meaning tailor) is the longest muscle in the body, with its origin insertion at the level of the anterior superior iliac spine (ASIS), running obliquely across the upper anterior third of the thigh in an inferomedial direction, with its distal insertion onto the anteromedial proximal tibia [2].

The sartorius muscle assists in flexion, abduction and lateral rotation of the hip [2, 3].

Muscle injuries occur in 31–46% athlete injuries, in comparison with ligament injuries, contusion, haematomas which constitute 18% of all injuries. Most upper thigh injuries include the adductor (23%), hamstring (12–37%) or quadriceps (19%) injuries, with the incidence of injuries increasing over the age of 30 years [4].

The pathogenesis of muscle injuries is divided into extrinsic and intrinsic injuries.

Extrinsic injuries include factors such as contusions and penetrating wounds, whereas intrinsic injuries are caused by contraction of elongation of the muscle, leading to destruction of the internal muscle fibre. Extrinsic injuries can involve all type of muscle fibres, whereas intrinsic injuries mainly involve type II muscle fibres which rapidly contract, extended between two joints, contract eccentrically and have a fusiform muscle fibre arrangement.

Intrinsic injuries are categorized into three grades based on the extent of the lesion: grade I involves a few muscle fibres within a bundle; grade 2 involves up to ¾ of the affected muscle portion; and grade 3 involves more than ¾, and the lesion may then involve the entire muscle belly [5].

The role of US is to assess the longitudinal extent of the lesion, calculate the volume of the haematoma and detect possible compression of surrounding anatomical structures [5].

Ultrasound examination of acute muscle injuries should be preceded by accurate reporting of the medical history, mechanism of injury as well as any other symptoms.

High-frequency ultrasound is an excellent method to assess both acute and chronic type muscle injuries. Sonographic assessment in real time along with correlation of clinical findings helps with diagnosis of muscle tears and the extent thereof.

Real-time palpation of the point of tenderness during sonographic evaluation enables a complimentary assessment during clinical evaluation. Dynamic evaluation of the muscle groups can also be useful [4].

### Case report

A 65-year-old male patient was referred to our department due to a suspected intrinsic muscular injury following extensive forceful traction of a heavy object along a short distance. No previous injury or trauma to the upper thigh was recorded nor any history of extraneous sporting event prior to the incident. He presented with a painful, palpable lump of the proximal third of the anterior upper thigh. Range of motion was limited with flexion, and lateral rotation of the hip. No visible ecchymosis was noted on the skin surface. The patient reported no use of oral anticoagulant therapy as well as no history of previous spontaneous bleeding.

### Ultrasound image evaluation

Ultrasound evaluation was an important tool to differentiate the various upper thigh muscle groups as well as to pinpoint to clinically evident lump on the anterior surface of the thigh. Longitudinal and transverse imaging was initiated from the upper knee joint to assess distal insertion of the quadriceps tendon. Transverse imaging during upward sweep along the thigh surface enabled viewing the vastus lateralis, medialis, intermedius as well as rectus femoris muscle bellies. The full extent of the quadriceps muscle was evaluated to exclude any secondary involvement. The proximal insertion of the quadriceps tendon was also evaluated by ultrasound and was confirmed intact.

**Fig. 1:**
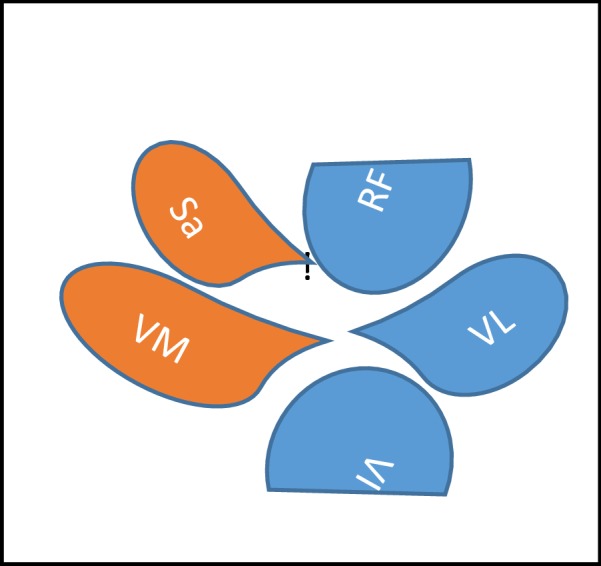
Schematic drawing of the anterior upper thigh. *Sa* Sartorius muscle, *RF* rectus femoris muscle, *VM* vastus medialis muscle, *VL* vastus lateral muscle, *VI*: vastus intermedius muscle

**Fig. 2:**
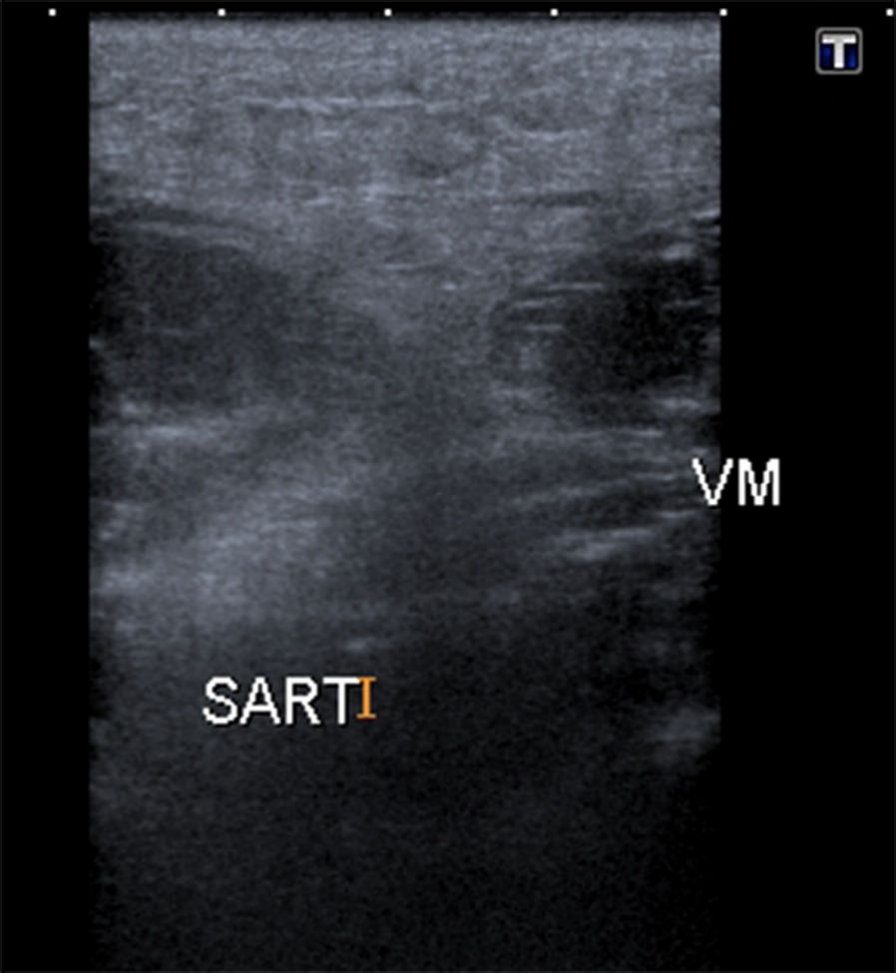
Transverse ultrasound image of sartorius muscle

The lump on the anterior surface of the thigh was confirmed as medial and superficial to the vastus medialis muscle group, which coincides with the anatomical position of the sartorius muscle along its inferomedial extent towards the medial tibial surface (see Figs. 1, 2).

**Fig. 3:**
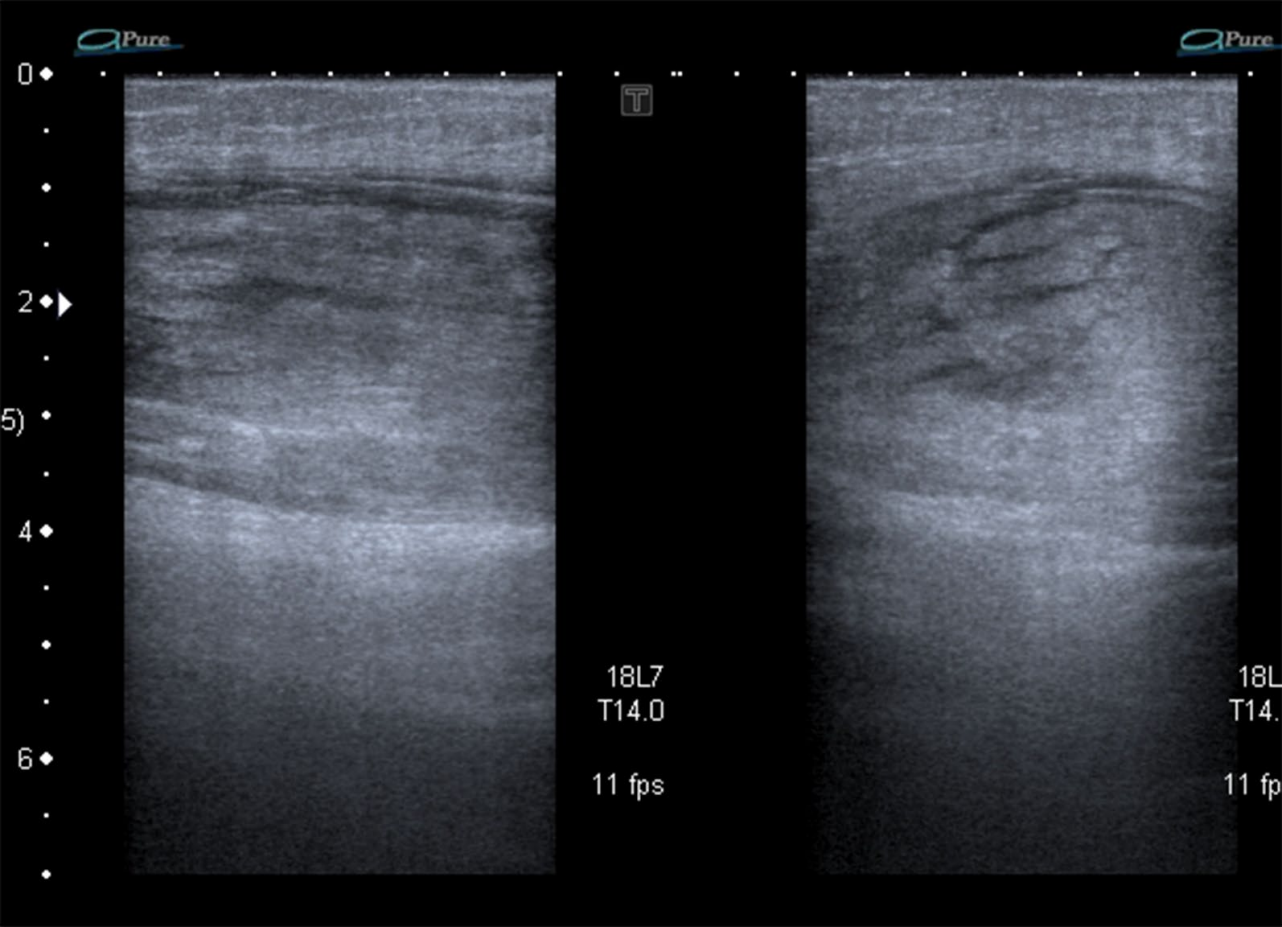
Hyperechoic, oedematous appearance in the sartorius muscle belly

**Fig. 4:**
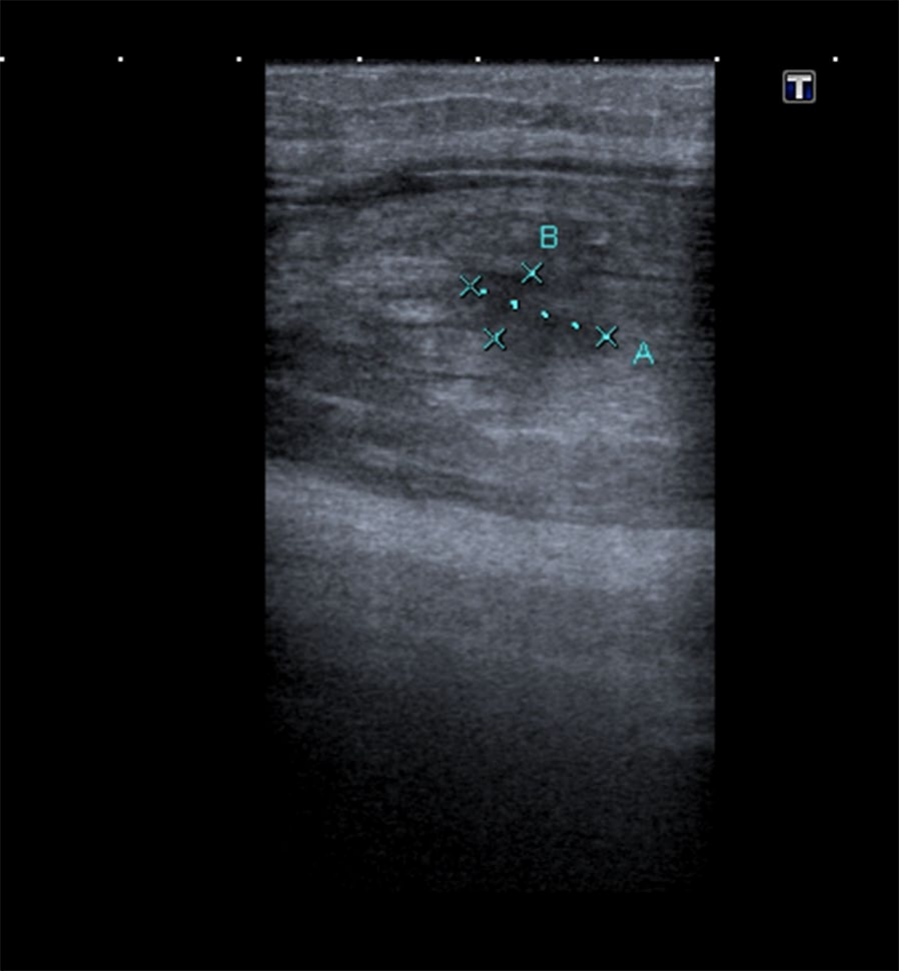
**a** Hypoechoic gap in the central muscle belly, solid arrow) with haematoma. **b** Schematic drawing of tear (black confirmed on ultrasound as intramuscular tear (Grade II)

**Fig. 5:**
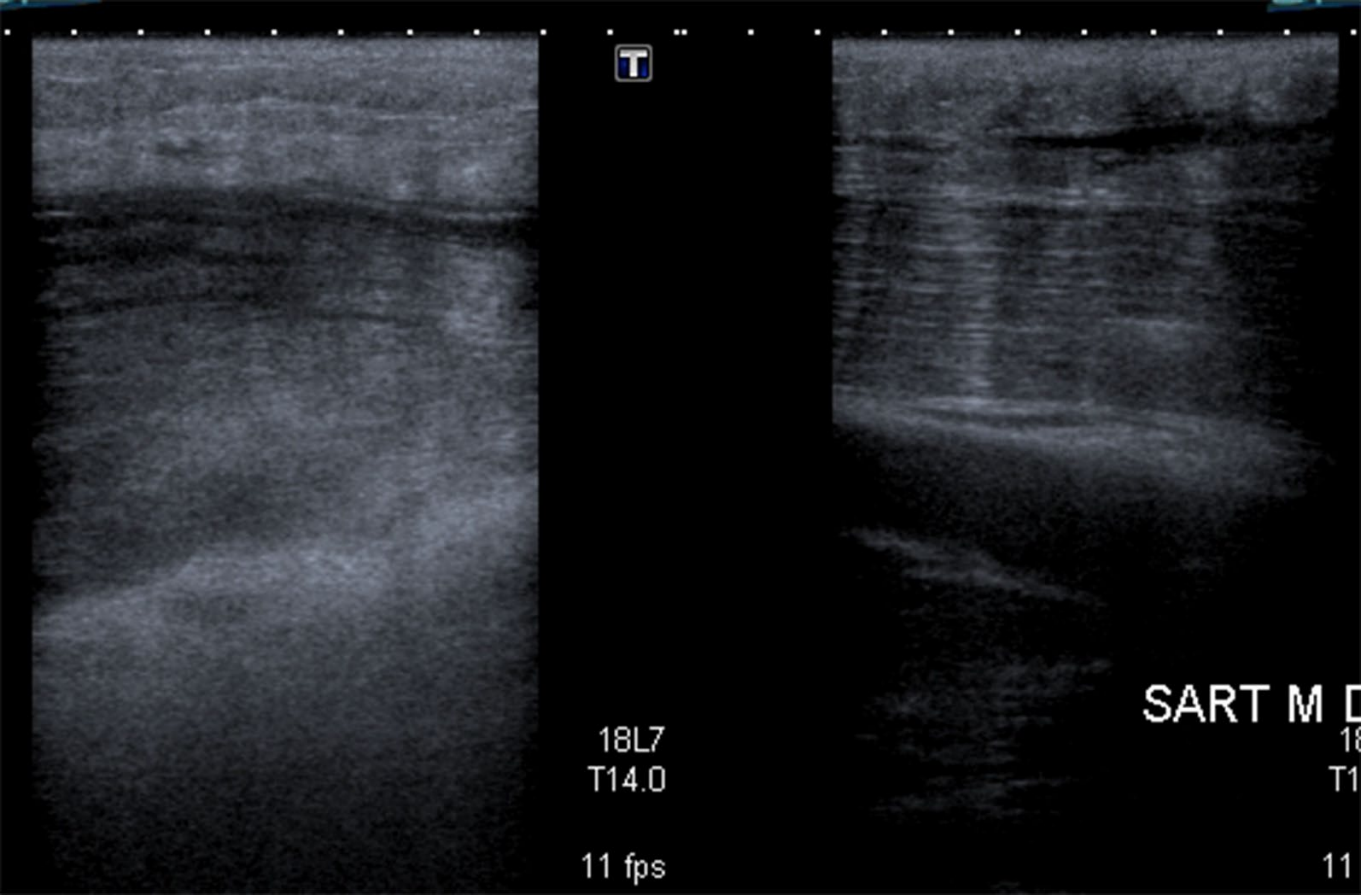
Loss of typical striated appearance of muscle at site of injury (dashed arrow), more distal to the injury site, the intact sartorius muscle has a typical striated muscular appearance (double arrow)

Increased hyperechoic, oedematous appearance of the sartorius muscle belly coincided with the palpable lump. Central to the oedematous swelling, there was a well-circumscribed hypoechoic fluid collection, which represents loss of typical muscular striations on ultrasound (blurring of the muscle bundles with local hypoechoic blood infiltration). This confirmed an intramuscular tear (grade II). See Figs. 3, 4, 5 and Table 1.

**Table 1:** US grading of intrinsic muscle injuries

Grade	US findings
0	No US image suggesting muscle injury
1	Small areas of damaged muscle tissue (< 5%)
2	Partial lesion (> 5–50%), which does not involve the entire muscle
3	Complete rupture of the muscle at the myotendinous junction

**Fig. 6:**
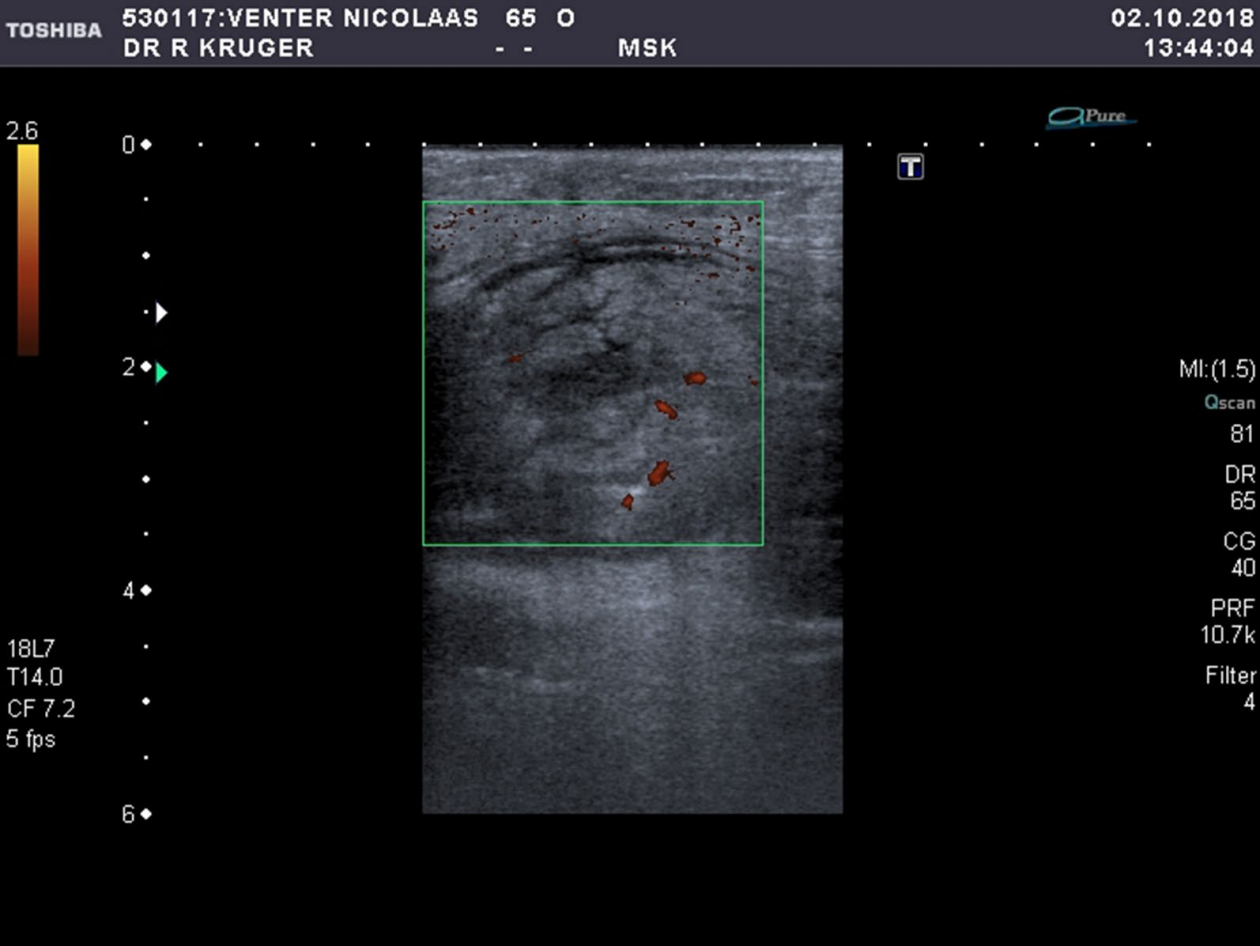
Increased vascularity noted with Power Doppler imaging

The surrounding hyperechoic oedematous muscle fibres confirmed haemorrhagic infiltration and was confirmed with power Doppler imaging as localized inflammatory reaction. See Fig. 6. It is important during the study of vascularity in muscle injury, to have correctly set parameters. Pulse repetition frequency (PRF) should be set to a maximum of 0.8 MHz, wall filter setting should be low, and the focus should be set on the area of interest to increase sensitivity for the detection of slow blood flow [1].

The extent of the tear was evaluated with longitudinal imaging for accurate measurement.

**Fig. 7:**
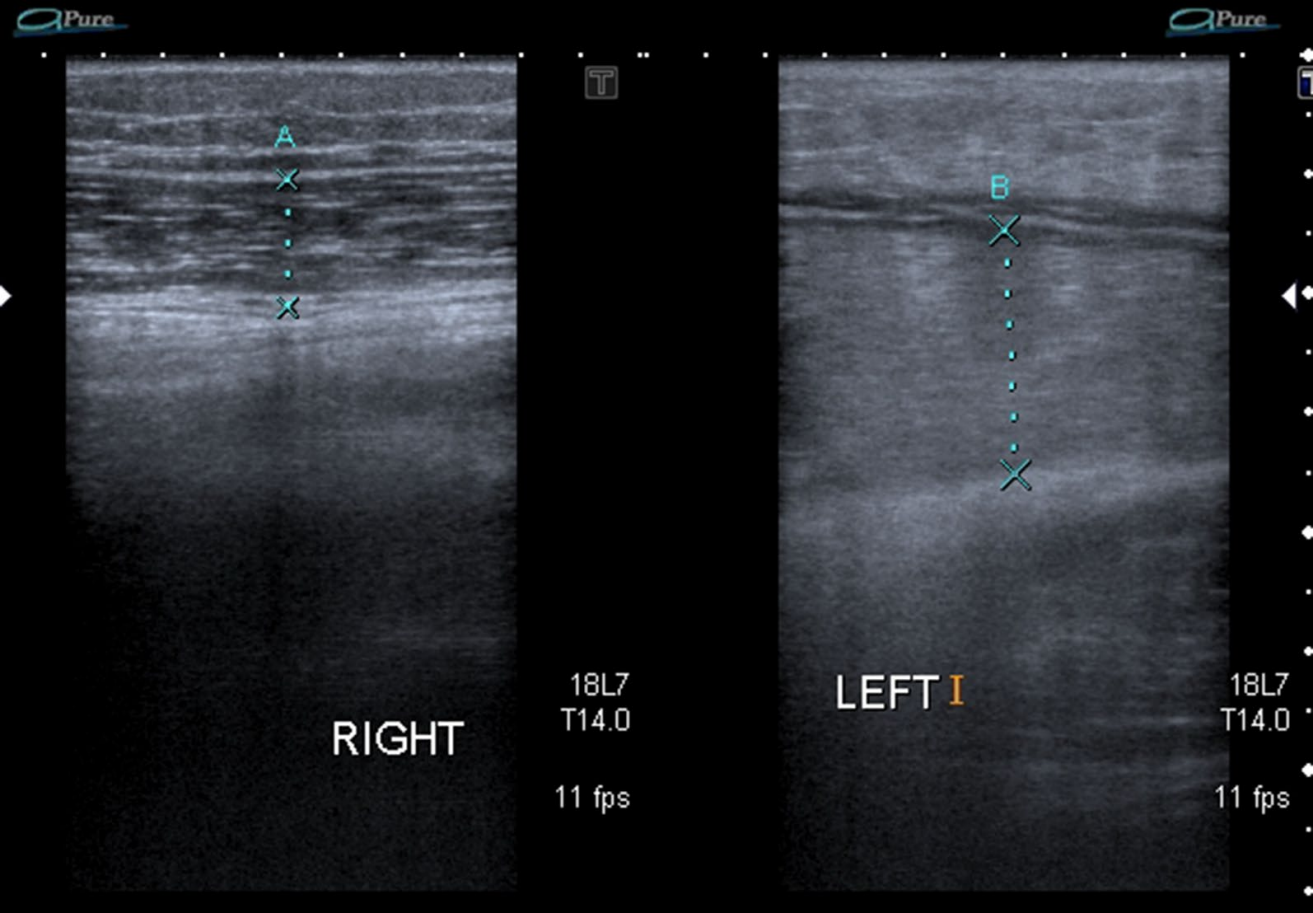
Comparison to the contralateral right side proves swelling and oedema of the affected leg

Comparison with the contralateral leg also aids in the evaluation process due to significant swelling in the acute phase of the affected leg. See Fig. 7.

## Discussion and conclusion

Intramuscular tears of the sartorius muscle in the acute phase are limited in the previous research, more specifically relating to the use of ultrasound as diagnostic tool. It is an uncommon site of injury and is considered as a deficit during the differential diagnosis of acute anterior hip pain [6].

In the elderly population, the use of anticoagulant therapy should be recorded as part of the initial clinical assessment, to determine the cause of any visible bleeding and or ecchymosis on the skin surface, following intrinsic injuries of the thigh.

Ultrasound imaging is essential for correct classification of an injury, assessment of severity and to exclude associated complications. US imaging proves useful in the acute phase as well as in the chronic phase to assess the healing process of muscle injury [1, 5].

The case is considered worthy of reporting to prove the important role of ultrasound in the radiological setting for diagnosis acute intramuscular tears in lieu of MRI imaging.

## Data Availability

Please contact author for data requests.
